# Brazilian Guidelines for Hereditary Angioedema Management - 2017 Update Part 1: Definition, Classification and Diagnosis

**DOI:** 10.6061/clinics/2018/e310

**Published:** 2018-04-21

**Authors:** Pedro Giavina-Bianchi, Luisa Karla Arruda, Marcelo V. Aun, Regis A. Campos, Herberto J. Chong-Neto, Rosemeire N. Constantino-Silva, Fátima R. Fernandes, Maria F. Ferraro, Mariana P.L. Ferriani, Alfeu T. França, Gustavo Fusaro, Juliana F.B. Garcia, Shirley Komninakis, Luana S.M. Maia, Eli Mansour, Adriana S. Moreno, Antonio A. Motta, João B. Pesquero, Nathalia Portilho, Nelson A. Rosário, Faradiba S. Serpa, Dirceu Solé, Priscila Takejima, Eliana Toledo, Solange O.R. Valle, Camila L. Veronez, Anete S. Grumach

**Affiliations:** I Divisao de Imunologia Clinica e Alergia, Hospital das Clinicas HCFMUSP, Faculdade de Medicina, Universidade de Sao Paulo, Sao Paulo, SP, BR; II Faculdade de Medicina de Ribeirao Preto, Universidade de Sao Paulo, Ribeirao Preto, SP, BR; III Departamento de Medicina Interna e Suporte Diagnostico, Faculdade de Medicina da Bahia, Salvador, BA, BR; IV Departamento de Pediatria, Universidade Federal do Parana, Curitiba, PR, BR; V Imunologia Clinica, Faculdade de Medicina do ABC, Santo Andre, SP, BR; VI Hospital do Servidor Publico Estadual Francisco Morato Oliveira, Sao Paulo, SP, BR; VII Divisao de Imunologia, Departamento de Medicina Interna, Universidade Federal do Rio de Janeiro, Rio de Janeiro, RJ, BR; VIII Departamento de Pediatria, Divisao de Imunologia Clinica e Alergia – Universidade Federal de Minas Gerais (UFMG), Minas Gerais, MG, BR; IX Divisao de Imunologia Clinica e Alergia, Departamento de Medicina Interna, Faculdade de Ciencias Medicas, Universidade Estadual de Campinas, Campinas, SP, BR; X Departamento de Biofisica, Universidade Federal de Sao Paulo, Sao Paulo, SP, BR; XI Escola Superior de Ciencias da Santa Casa de Misericordia de Vitoria, Vitoria, ES, BR; XII Divisao de Alergia, Imunologia e Reumatologia Clinica, Departamento de Pediatria, Universidade Federal de Sao Paulo, Sao Paulo, SP, BR; XIII Divisao de Alergia e Imunologia Clinica, Faculdade de Medicina de Sao Jose do Rio Preto, Sao Jose do Rio Preto, SP, BR

**Keywords:** Hereditary Angioedema, Angioedema, C1 Inhibitor Deficiency, Coagulation Factor XII Mutations, Management, Guidelines

## Abstract

Hereditary angioedema is an autosomal dominant disease characterized by recurrent angioedema attacks with the involvement of multiple organs. The disease is unknown to many health professionals and is therefore underdiagnosed. Patients who are not adequately diagnosed and treated have an estimated mortality rate ranging from 25% to 40% due to asphyxiation by laryngeal angioedema. Intestinal angioedema is another important and incapacitating presentation that may be the main or only manifestation during an attack. In this article, a group of experts from the “Associação Brasileira de Alergia e Imunologia (ASBAI)” and the “Grupo de Estudos Brasileiro em Angioedema Hereditário (GEBRAEH)” has updated the Brazilian guidelines for the diagnosis and treatment of hereditary angioedema.

## WHY STUDY HEREDITARY ANGIOEDEMA?

Since the first Brazilian Hereditary Angioedema Guidelines were published in 2011, the body of knowledge regarding hereditary angioedema (HAE) has increased, and its management has improved 1-6. More patients have been diagnosed with HAE, and new treatments for the disease have become available in our country. In this article, a group of experts in HAE from the Brazilian Association of Allergy and Immunology (Associação Brasileira de Alergia e Imunologia - ASBAI) and the Hereditary Angioedema Brazilian Study Group (Grupo de Estudos Brasileiro em Angioedema Hereditário - GEBRAEH) have updated the guidelines for the diagnosis and therapy of hereditary angioedema.

Prof. William Osler, who established the hereditary nature of HAE, stated that “Medicine is both a science and an art”. Developing and applying evidence-based guidelines is fundamental for the practice of medicine as a science; however, assisting individual patients with their biopsychosocial features in a personalized manner is practicing medicine as an art.

HAE remains unknown to and underdiagnosed by many health care providers. The long periods from the onset of symptoms to the diagnosis of the disease and from diagnosis to access to therapy increase HAE-related morbidity and mortality, thus affecting the quality of life (QoL) of patients and their families 1,2,5-9. HAE patients visit an average of 4.4 physicians before receiving a correct diagnosis, and 65% of these patients are misdiagnosed 10,11. Although HAE accounts for a minority of cases of angioedema, and the initial estimate of its prevalence was 1:50,000 (ranging from 1:10,000 to 1:150,000), new subsets of patients with the disease have been described; thus, the disease is more common than previously thought 6,12-15. Physicians and other health professionals should be aware of the clinical presentation of HAE and of screening laboratory tests whose results may be suggestive of its diagnosis. In addition, allergy and immunology specialists must be updated with information on the diagnosis and management of patients with HAE.

The two most severe clinical manifestations of HAE are related to the upper airways and intestinal angioedema. Patients who are not properly treated have estimated mortality rates of 25% to 40% due to asphyxiation by laryngeal angioedema 3,5,12,16. In the United States, HAE accounts for 15,000-30,000 emergency room visits per year. These visits often lead to hospitalization and admission to intensive care units 17,18. Incapacitating intestinal angioedema is another major manifestation of HAE, and the intestine can be the main or only organ system involved in an attack. Such patients are often misdiagnosed as having an acute surgical abdomen and undergo unnecessary surgeries 12,19,20. It is estimated that patients with HAE experience some degree of disability for 20-100 days per year 4,9.

## WHAT IS HEREDITARY ANGIOEDEMA?

Angioedema is a localized, self-limiting, asymmetric and disfiguring non-inflammatory edema of the deep dermis or subcutaneous or submucosal tissues that occurs as a result of vasodilation and increased vascular permeability. HAE comprises a group of diseases characterized by recurrent angioedema caused by excess bradykinin production, with an autosomal dominant inheritance pattern.

## WHAT ARE THE TYPES OF HEREDITARY ANGIOEDEMA?

In an attempt to standardize nomenclature, an international panel of HAE experts issued a consensus statement in which they proposed a classification for “Angioedema without wheals” based mainly on the presence or absence of C1-INH deficiency 4. The following three forms of HAE have been defined:

### 1) HAE with quantitative C1-INH deficiency (previously designated C1-INH-HAE Type-I)

HAE characterized by a quantitative decrease in C1-INH expression to levels below 50% of the normal value and, consequently, a decrease in functional protein activity. This phenotype is the most prevalent form of HAE (80-85% of cases of the disease are caused by C1-INH deficiency) 21.

### 2) HAE with C1-INH dysfunction (previously designated C1-INH-HAE Type-II)

HAE in which C1-INH levels are normal or elevated, but the function of the protein is impaired 22.

### 3) HAE with normal C1-INH (previously designated HAE Type III)

This form of HAE, which was identified more recently than the other forms of the disease, affects mainly women but has also been identified in men. This form is characterized by clinical symptoms similar to those of HAE with C1-INH deficiency, along with heritability and normal C1-INH levels and function 15,23. HAE with normal C1-INH has been associated with increased serum estrogen levels (pregnancy and exogenous administration) and mutations in the gene encoding FXII in a subset of patients (FXII-HAE). However, in a significant percentage of cases, the genetic defect remains unknown. Therefore, the following two subtypes have been recognized: HAE with normal C1-INH and FXII mutation (FXII-HAE) and HAE with normal C1-INH and unknown genetic defect (U-HAE) 1,2,5-9,24. It has been recommended that clinicians no longer use the term HAE type III because it is not associated with C1-INH deficiency.

## WHAT IS THE CAUSE OF HEREDITARY ANGIOEDEMA?

Quincke first described HAE as a clinical entity in 1882 25-27, and Osler established its hereditary nature in 1888 28-32. The first biochemical change associated with the disease, C1 inhibitor (C1-INH) deficiency, was not identified until 75 years later. Patients with HAE due to C1-INH deficiency present with a quantitative or qualitative defect of the protein, a serine protease inhibitor from the SERPIN superfamily 33-35. The protein inhibits the first components of the complement system-classical pathway, C1r and C1s esterases, which bind to and activate C1q 36. In the absence of such inhibition, the complement system becomes excessively activated. C1-INH has also been recognized as an inhibitor of several proteases, including plasma kallikrein, coagulation factors XII (FXII) and XI, plasmin and MASP-1 and MASP 2, which are components of the complement system-lectin pathway. Therefore, C1-INH inhibits the complement system and participates in regulating the contact, coagulation and fibrinolysis systems 37-39.

**Figure 1:**
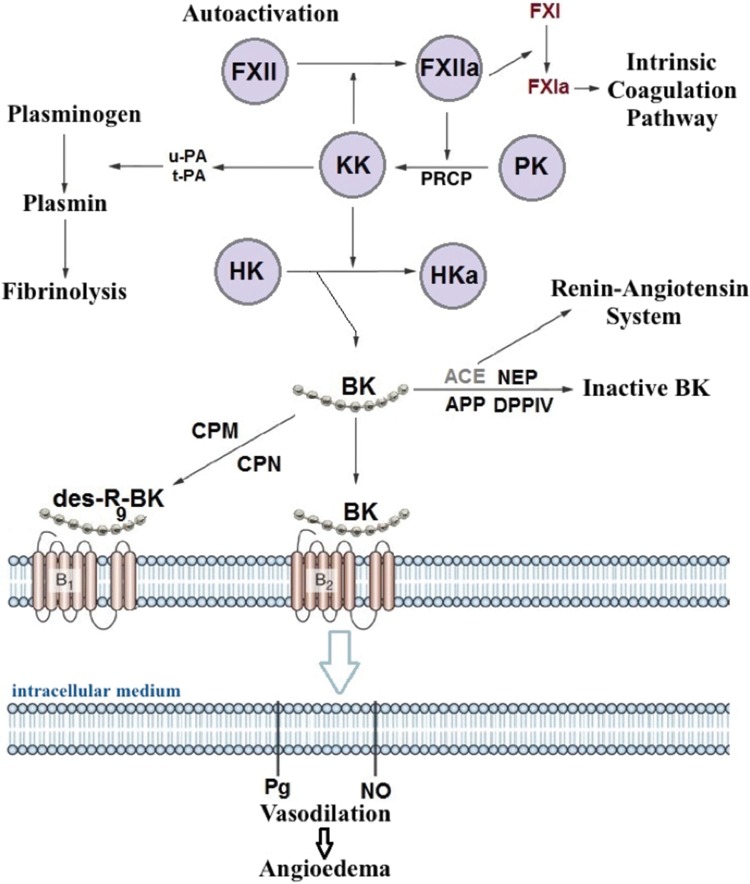
Kallikrein-kinin system.

Episodes of angioedema were initially attributed to factors formed during complement system activation, including a C2 fragment (C2 kinin) associated with vasodilation and increased permeability. Subsequent studies have revealed that C1-INH deficiency results in over-activation of the contact system (kallikrein-kinin system) and increased bradykinin production (Figure 1). Bradykinin binds to its B2 receptor, which is constitutively expressed on endothelial cells, and decreases endothelial junctions, thereby increasing vascular permeability and inducing angioedema 40,41. Bradykinin also stimulates nitric oxide production, leading to vasodilation induced by contraction of the cytoskeleton 42. Evidence indicates that bradykinin is the main HAE mediator 43,44.

In 1999, Nussberger et al. assessed plasma samples from patients with HAE and observed that bradykinin levels were higher in blood drawn from edematous sites than in blood drawn from other sites that were not affected by the disease 45. Another study showed that vascular permeability was lower in knockout mice with concomitant C1-INH and bradykinin receptor B2 (BDKRB2) deficiencies than in mice with only C1-INH deficiency, demonstrating that the bradykinin/BDKRB2 pathway plays an important role in angioedema 46. The investigation and development of new treatments, such as bradykinin receptor antagonist and kallikrein inhibitors, has reinforced the role of bradykinin as the main mediator of HAE 47,48.

In conclusion, HAE with C1-INH deficiency occurs due to decreased levels of the protein or as a consequence of the production of a dysfunctional protein. Among patients with HAE, the levels of functional C1-INH typically range from 5% to 30% of the normal value. One would expect the functional level of the protein to reside at approximately 50% of the normal value because almost all patients are heterozygous for the mutation and thus bear a functioning allele of the C1-INH gene 4,12. The reasons for the lower-than-expected functional activity of C1-INH remain unclear. The above findings suggest that additional mechanisms may underlie HAE development.

In 2000, Bork et al. identified a new subset of patients with HAE with normal C1-INH levels 15. Mutations of the gene encoding coagulation factor XII (FXII), which segregated within the families of patients with HAE with normal C1-INH levels, were subsequently described. This HAE type was designated FXII-HAE 49. FXII plays a central role in the early stages of contact system activation by increasing bradykinin synthesis 50. Initial observations suggested that mutations in FXII lead to gain-of-function changes and subsequent increases in bradykinin production; however, subsequent studies have failed to confirm these earlier findings, and the functional role of the observed mutations remains unclear 51,52. By generating recombinant natural and mutated variants of FXII, studies have recently shown that FXII-HAE mutations introduce new sites that are sensitive to enzymatic cleavage by plasmin, thus rendering FXII mutants abnormally sensitive to plasmin. The FXII mutants rapidly activate after cleavage by plasmin and escape from inhibition through C1-INH, leading to excessive bradykinin production. Interestingly, the authors provided evidence that lysine analogs, including tranexamic acid and epsilon aminocaproic acid, may attenuate these changes. This finding explains the therapeutic value of these agents in patients with FXII-HAE 53. However, not all patients with HAE with normal C1-INH levels bear a mutation in FXII and, thus, have HAE of unknown cause (U-HAE) 24. As is the case in HAE with C1-INH deficiency, HAE with normal C1-INH levels appears to be mediated by bradykinin.

Patients with HAE do not present urticaria, experience worsening symptoms with angiotensin-converting enzyme inhibitors (ACEi) and estrogen treatments, fail to respond to antihistamines and corticosteroids and improve with the use of the bradykinin receptor B2 blocker 4,23. Affected patients with HAE, especially those with normal C1-INH levels, show worsening symptoms with exogenous estrogen intake, such as that which occurs during contraception or hormone replacement therapy 23,54. The mechanisms underlying this deterioration are only partially understood. The promoter region of the FXII gene contains an estrogen responsive element, and increases in FXII mRNA transcription in response to estrogen have been demonstrated 55. It is likely that estrogen also contributes to the regulation of B2 bradykinin receptor expression, modulates the kallikrein-kinin cascade and reduces C1-INH levels 56,57.

## WHAT ARE THE TYPICAL CLINICAL MANIFESTATIONS OF HEREDITARY ANGIOEDEMA?

The clinical history is an important component of the diagnosis of HAE, and it has been well characterized in the cohorts studied by Profs. Agostoni, Cicardi and Bork 2,3,5,11. Patients with HAE suffer from recurrent attacks of edema involving the skin and submucosa of several organs. HAE is not associated with urticaria and is nonpruritic, but patients sometimes describe experiencing a burning sensation in edematous regions. The sites most commonly affected are the face, extremities, genitalia, oropharynx, larynx and digestive system. However, rare clinical manifestations of the disease, such as intense headaches caused by brain edema, urinary retention and acute pancreatitis, may also occur 58.

The frequency and severity of the above clinical manifestations vary among individuals. It has been reported that 5% of patients with HAE are asymptomatic and that 25% develop sporadic symptoms 5,6,13,15. A retrospective study analyzing 131,110 attacks in 221 patients with HAE reported that laryngeal edema occurred in less than 1% of attacks, although over 50% of patients enrolled in the study had previously experienced this particular manifestation at least once 5.

HAE attacks typically last 48-72 hrs if left untreated and do not improve with administration of antihistamines, corticosteroids or epinephrine. Although many attacks occur spontaneously, several factors that trigger them have been identified, including minor trauma, stress, infection, menstruation, pregnancy, alcohol consumption, extreme temperature changes, exercise, ACEi use and estrogen use (contraceptives and hormone replacement therapy) 2,3,5,11.

In some patients, erythema *serpiginous* can occur as a prodromal manifestation preceding an HAE attack, but the concomitant presence of pruritic urticaria favors the diagnosis of histaminergic angioedema, making the diagnosis of HAE unlikely 5. Nevertheless, some cases of HAE accompanied by urticaria have been reported. In addition to the above phenomenon, irritability, weakness, nausea and “flu sensations” have also been reported as prodromal manifestations of the disease.

During adolescence, patients may experience substantial increases in disease activity. In particular, girls may experience increases in disease activity due to menstruation or the use of contraceptives containing estrogen. A family history of angioedema is suggestive of a diagnosis of HAE; however, this history may be absent in up to one-fourth of affected patients.

**Figure 2:**
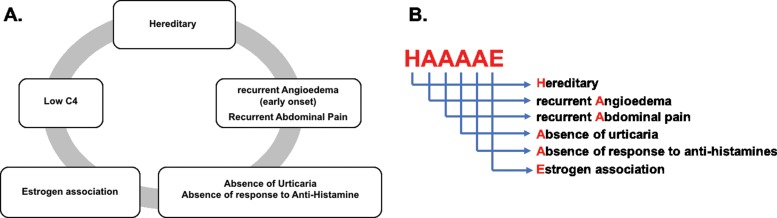
A: Warning Signs. B: HAAAAE for Heredity, recurrent Angioedema, recurrent Abdominal pain, Absence of urticaria, Absence of response to antihistamines and association with Estrogen.

Although the clinical manifestations of HAE with normal C1-INH are similar to those of other types of HAE, there are some differences between them. Symptom onset generally occurs later in this type of HAE, and the course of the disease tends to be more benign than that of others. Moreover, tongue involvement is common. Bruising is occasionally seen at sites affected by angioedema. However, the most striking characteristic of HAE with normal C1-INH is its association with female gender and estrogen intake 24. We have generated a list of warning signs of the disease and have also devised an acronym to increase awareness of HAE. These items are presented in Figure 2.

## HOW CAN LABORATORY TESTS CONFIRM THE DIAGNOSIS OF HEREDITARY ANGIOEDEMA?

**Figure 3:**
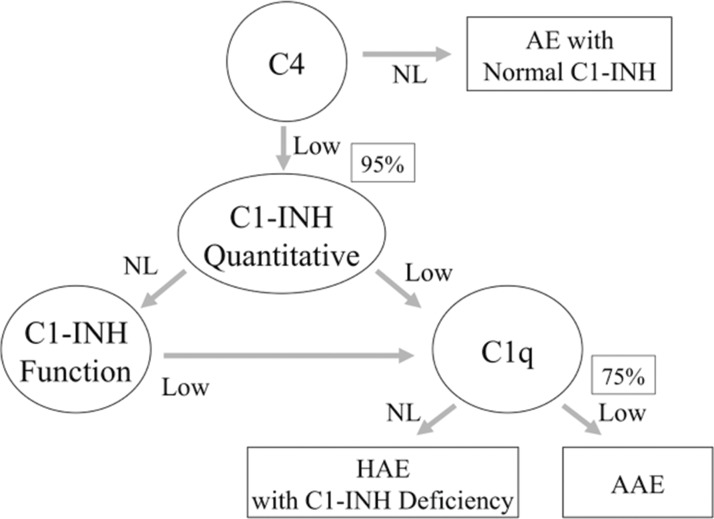
Algorithm of HAE diagnosis.

Individuals clinically suspected of having HAE and those with a family history of HAE should be investigated (Figure 3). Serum C4 levels can be used as a screening test because quantitative or qualitative C1-INH deficiency leads to permanent complement system activation, which is accompanied by C4 consumption, even when patients are not experiencing an angioedema attack. C4 levels normalize during the inter-crisis period in only 2-5% of patients with HAE 3,5,10,16. However, determining C3 levels is unnecessary, as C3 levels are normal in patients with HAE because C3 has greater turnover than C4, and C1-INH does not directly regulate its activation. Additionally, C3 levels and activity are regulated by other factors, such as factors H and I from the alternative complement pathway.

In addition to performing measurements of serum C4 levels, quantitative and functional evaluations of C1-INH should be performed. All health professionals and family members involved in providing care for patients with HAE must ensure that such tests are available. Although quantitative determination of C1-INH levels is relatively easy, evaluations of functional C1-INH activity (qualitative tests) must be performed at referral laboratories 12,14,18. Ideally, these tests should be performed immediately after sample collection to avoid degradation. However, as this is not feasible in most cases, reliable test results can be achieved when samples are properly stored and the tests performed with adequate methodology. When using the chromogenic functional assay, it is critical that samples are kept at -20°C in all steps of the process, including storage and transportation, for accurate results 59,60. It is mandatory to avoid freezing and thawing the same sample more than once for functional C1-INH evaluation. A functional activity test is usually performed only when the quantitative determination of C1-INH is normal (Figure 3). However, some studies suggest that functional activity could be a suitable screening test in addition to C4 level measurement, considering that functional activity would be decreased in all patients with HAE due to C1-INH deficiency (types I and II) 60.

If the clinical suspicion of HAE due to C1-INH deficiency remains in the presence of normal C4 levels, the test should be repeated during an episode of angioedema whenever possible, as C4 levels are occasionally (2-5%) normal between attacks 61. If the test result is again normal, and quantitative and qualitative levels of C1-INH are normal, a diagnosis of HAE with normal C1-INH is suggested, a condition in which all of these biochemical parameters are normal 17.

Analysis of *SERPING1,* the gene encoding C1-INH, may be performed in cases of undefined diagnoses or for research purposes. Mutations can be identified in one of the eight exons or exons/introns adjacent regions of the gene, which affect the production of the protein and/or its function. Not all mutations detected by routine genetic testing are disease-causing, and genetic testing of other affected and disease-free family members is sometimes needed. Genotyping may be recommended whenever a discrepancy exists between a patient's clinical history and laboratory test results and in newborns or infants or specific situations, such as when patients with late-onset disease or no family history of disease present with clinical symptoms suggestive of HAE 62,63.

Factor XII gene mutations have been identified in a subset of patients with HAE with normal C1-INH. Initially, genetic sequencing detected two different missense mutations in exon 9: one encodes a threonine-to-lysine substitution (p.Thr328Lys), and the other encodes a threonine-to-arginine substitution (p.Thr328Arg) 49. Novel mutations have subsequently been published: a mutation featuring a deletion of 72 base pairs (c.971_1018+24del72), which was reported in a family originating from Turkey; and a mutation featuring a duplication of 18 base pairs (c.892_909dup), which has been described in patients from Hungary 64,65.

In acquired angioedema with C1-INH deficiency (C1-INH AAE), a condition associated with lymphoproliferative or autoimmune diseases, paraproteins induce the activation and consumption of complement components. Therefore, determining C3 and C1q levels, the latter of which are reduced in 75% of patients, may aid clinicians in differentiating between HAE and AAE. In addition to C1q consumption, anti-C1-INH antibodies may be observed in AAE associated with autoimmune disease 66.

## WHAT ARE THE DIAGNOSTIC CRITERIA FOR HAE?

Criteria with which the diagnosis of HAE with C1-INH deficiency can be standardized have been proposed (Table 1). According to those criteria, the diagnosis of HAE is confirmed when patients meet one primary clinical and one biochemical disease criterion 12,19,20. It should be highlighted that the criteria are not absolute and that clinical history takes precedence over laboratory findings, particularly in localities in which laboratory tests are not available. In selected cases, a therapeutic test can aid in establishing the diagnosis of HAE.

The diagnosis of HAE with normal C1-INH is considered in patients with recurrent angioedema not associated with urticaria and with normal C1-INH activity and plasma protein levels. A history of one or more family members also being affected by the disease, along with a history of the disease occurring predominantly in female family members, makes the diagnosis more likely. At this time, the only confirmatory test for HAE with normal C1-INH is the study of the *F12* gene to identify one of the mutations that has been described (FXII-HAE) 9,17.

## WHAT IS NOT HEREDITARY ANGIOEDEMA?

**Figure 4:**
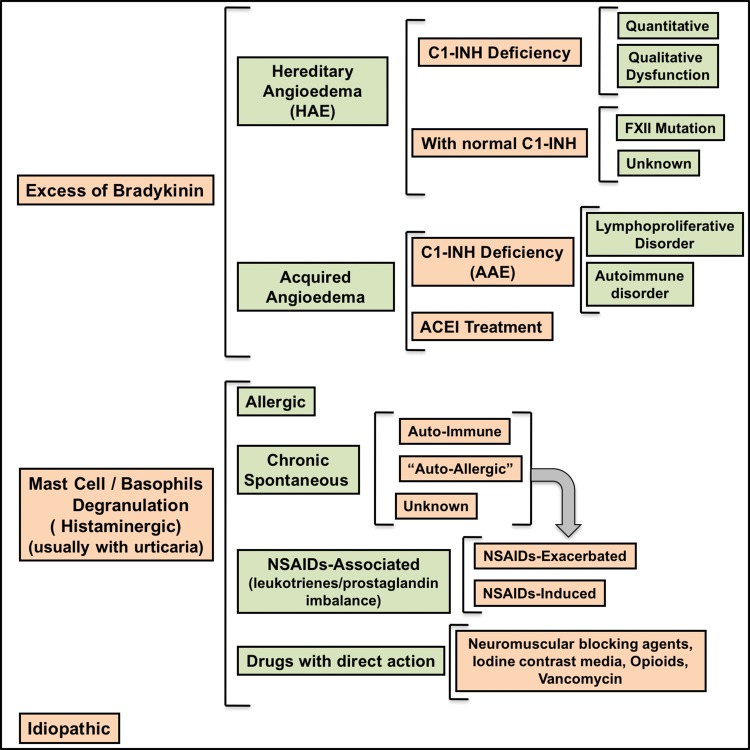
Classification of Angioedema by Endotypes 25,27.

Two major pathophysiological mechanisms underlying the development of angioedema have been described. Angioedema can be induced by the activation of mast cells and/or basophils, resulting in the release of histamine and other mediators (histaminergic angioedema), and by an excess of bradykinin (bradykinin-mediated or non-histaminergic angioedema), as seen in HAE, acquired angioedema with C1-INH deficiency (AAE) and angioedema induced by ACEi and other drugs involved in bradykinin metabolism 4,29. A scheme in which angioedema is classified according to endotype has recently been proposed and may provide clinicians with insights into the etiology of the disease and/or the pathophysiological mechanism underlying the development of the different types of this clinical entity (Figure 4) 25,27,33-35.

The differential diagnosis for HAE encompasses other types of angioedema, mainly those with chronic or recurrent presentations. The most frequent type of recurrent angioedema is the histaminergic type, which is usually associated with urticaria. This type can be induced or exacerbated by the use of non-steroidal anti-inflammatory drugs (NSAIDs) 28,30-32,36. The presence of urticaria, the improvement of the disease with the administration of antihistamines, and the triggering of symptoms by NSAIDs intake make the diagnosis of HAE less likely in a patient with angioedema. However, chronic histaminergic angioedema can present without urticaria, and NSAIDs are one of the major causes of angioedema without urticaria 25. In contrast, it is not necessary to avoid prescribing NSAIDs for patients with HAE, as the pathophysiological mechanisms underlying the development of angioedema are different.

The current guidelines for treating chronic spontaneous urticaria/angioedema highlight the fact that some patients will not respond to conventional doses of antihistamines and may need their dose increased to levels as high as four times the recommended daily doses to control their symptoms. Therefore, a trial with an antihistaminic agent administered at four times the recommended dose is warranted. This trial must last for a period of time sufficient to determine whether the angioedema has been controlled to confirm or rule out its histaminergic nature. The safety of up-dosing antihistamines, including cetirizine, levocetirizine, desloratadine, fexofenadine and rupatadine, has been documented in previous studies 67.

It is very important to ask patients about the use of ACEi when attempting to diagnose acquired forms of bradykinin-mediated angioedema. As ACE is the major enzyme involved in bradykinin degradation, its inhibition leads to increased serum concentrations of this mediator and may cause angioedema. ACEi should be withdrawn in all patients with recurrent angioedema, even if the angioedema developed several years after treatment onset. Up to 0.7% of individuals who take ACEi present with recurrent angioedema, and the risk of the disease is increased among African-descendants, smokers, elderly patients and females 68,69. ACEi-induced angioedema more frequently involves the face, tongue, neck and larynx than other sites; however, sporadic cases of abdominal attacks have been reported. The mean time for the onset of angioedema symptoms is 1.8 years after treatment initiation; however, symptoms occur in the first month post-treatment initiation in 25% of cases and may occur up to 10 years after ACEi treatment initiation 70. Although symptoms of angioedema induced by ACEi may resemble those of HAE, affected patients will have normal C4 and C1q levels and normal C1-INH levels and function. More rarely, angiotensin II receptor antagonists (ARBs) and gliptins, which are oral hypoglycemic agents that inhibit dipeptidyl peptidase IV, another enzyme involved in bradykinin catabolism, may also induce angioedema 71.

AAE is another form of angioedema characterized by C1-INH deficiency (C1-INH AAE); however, this disease is not inherited. In C1-INH AAE, the onset of symptoms occurs later, there is no family history of angioedema and the disease is caused by the consumption of C1-INH or the production of C1-INH-neutralizing autoantibodies, which are associated with lymphoproliferative disorders or autoimmune diseases, respectively 12,72,73. As a consequence, C1-INH activity is low, the complement system is activated and C1q levels are usually decreased, a particular feature that can aid disease diagnosis 74,75. Moreover, C1-INH function decreases to levels less than 50% of normal, and C1-INH antigen levels are generally decreased, although the presence of cleaved C1-INH may result in apparently normal C1-INH antigen levels in approximately 20% of patients 74,76. C1-INH AAE with autoantibodies and C1-INH AAE with lymphoproliferative diseases largely overlaps and should be considered the same disease 4.

Idiopathic non-histaminergic angioedema is characterized as non-hereditary and is diagnosed in cases in which all known causes of angioedema have been excluded. Its symptoms persist despite treatment with high doses of antihistamines, doses that are typically as high as 4 times the standard doses of second-generation, non-sedating antihistamines. Some evidence exists indicating that bradykinin may be the mediator involved in the development of idiopathic non-histaminergic angioedema; however, this evidence is not definitive, as other vasoactive mediators, including cysteinyl-leukotrienes, prostaglandins and platelet-activator factor, may play a role in the development of the disease in some patients. Treating patients with idiopathic non-histaminergic angioedema is often a challenge in clinical practice, and no definitive recommendations for the treatment of such patients are reliable. The capacity of tranexamic acid and icatibant to alleviate symptoms has been reported in individual cases. Cyclosporine and omalizumab have also been used to treat affected patients, indicating that this group of patients is heterogeneous. We speculate that this classification may include patients with HAE with normal C1-INH levels and without a family history of the disease and patients with chronic spontaneous urticaria/angioedema resistant to antihistamines, given that up to 10% of these patients present with recurrent angioedema without urticaria 4,27,67.

**Conflicts of Interest.** The authors PGB, MVA, RGA, HJCN, MPLF, JFBG, EM, ET, SORV and ASG declare that they have received financial support from CSL Boehring and Shire. The authors LKA, MFF, LSMM, AAM, ASM and FSS declare that they have received financial support from Shire. The authors RNCS, FRF, ATF, GF, SK, JBP, NP, NAR, DS, PT and CLV have no conflicts of interest to declare.

## AUTHOR CONTRIBUTIONS

All authors meet the following three conditions: 1) All authors made substantial contributions to the conception of the study; 2) All authors contributed to drafting the manuscript and its critical revision for important intellectual content; 3) All authors provided final approval of the version to be published.

## Figures and Tables

**Table 1:** Diagnostic Criteria for Hereditary Angioedema with C1-INH Deficiency 12,26.

**I – Primary clinical criteria**
a) **Non-inflammatory subcutaneous angioedema lasting longer than 12 hrs**
b) **Abdominal pain with no specific etiology lasting longer than 6 hrs**
**c) Laryngeal edema**
**II – Secondary clinical criteria**
a) **Family history of hereditary angioedema**
**III – Biochemical criteria**
a) **Quantitative C1-INH deficiency (<50%, in two distinct samples)**
b) **Functional C1-INH deficiency (<50%, in two distinct samples)**
c) **Mutation in the gene encoding C1-INH (*SERPING1*)**

## References

[1] Giavina-Bianchi P, França AT, Grumach AS, Motta AA, Fernandes FR, Campos RA (2011). Brazilian guidelines for the diagnosis and treatment of hereditary angioedema. Clinics.

[2] Agostoni A, Cicardi M (1992). Hereditary and acquired C1-inhibitor deficiency: biological and clinical characteristics in 235 patients. Medicine (Baltimore).

[3] Cicardi M, Bergamaschini L, Marasini B, Boccassini G, Tucci A, Agostoni A (1982). Hereditary angioedema: an appraisal of 104 cases. Am J Med Sci.

[4] Cicardi M, Aberer W, Banerji A, Bas M, Bernstein JA, Bork K (2014). Classification, diagnosis, and approach to treatment for angioedema: consensus report from the Hereditary Angioedema International Working Group. Allergy.

[5] Bork K, Meng G, Staubach P, Hardt J (2006). Hereditary angioedema: new findings concerning symptoms, affected organs, and course. Am J Med.

[6] Craig T, Aygören-Pürsün E, Bork K, Bowen T, Boysen H, Farkas H (2012). WAO Guideline for the management of hereditary angioedema. World Allergy Organ J.

[7] Gomide MA, Toledo E, Valle SO, Campos RA, França AT, Gomez NP (2013). Hereditary angioedema: quality of life in Brazilian patients. Clinics.

[8] Prior N, Remor E, Pérez-Fernández E, Caminoa M, Gómez-Traseira C, Gayá F (2016). Psychometric field study of hereditary angioedema Quality of Life Questionnaire for adults: HAE-QoL. J Allergy Clin Immunol Pract.

[9] Lumry WR, Castaldo AJ, Vernon MK, Blaustein MB, Wilson DA, Horn PT (2010). The humanistic burden of hereditary angioedema: impact on health-related quality of life, productivity, and depression. Allergy Asthma Proc.

[10] Farkas H, Varga L, Széplaki G, Visy B, Harmat G, Bowen T (2007). Management of hereditary angioedema in pediatric patients. Pediatrics.

[11] Lunn ML, Santos CB, Craig TJ (2010). Is there a need for clinical guidelines in the United States for the diagnosis of hereditary angioedema and the screening of family members of affected patients. Ann Allergy Asthma Immunol.

[12] Agostoni A, Aygören-Pürsün E, Binkley KE, Blanch A, Bork K, Bouillet L (2004). Hereditary and acquired angioedema: problems and progress: proceedings of the third C1 esterase inhibitor deficiency workshop and beyond. J Allergy Clin Immunol.

[13] Longhurst HJ, Farkas H, Craig T, Aygören-Pürsün E, Bethune C, Bjorkander J (2010). HAE international home therapy consensus document. Allergy Asthma Clin Immunol.

[14] Bowen T, Cicardi M, Farkas H, Bork K, Longhurst HJ, Zuraw B (2010). International consensus algorithm for the diagnosis, therapy and management of hereditary angioedema. Allergy Asthma Clin Immunol.

[15] Bork K, Barnstedt SE, Koch P, Traupe H (2000). Hereditary angioedema with normal C1-inhibitor activity in women. Lancet.

[16] Bork K, Hardt J, Schicketanz KH, Ressel N (2003). Clinical studies of sudden upper airway obstruction in patients with hereditary angioedema due to C1 esterase inhibitor deficiency. Arch Intern Med.

[17] Bork K (2010). Diagnosis and treatment of hereditary angioedema with normal C1 inhibitor. Allergy Asthma Clin Immunol.

[18] Moore GP, Hurley WT, Pace SA (1988). Hereditary angioedema. Ann Emerg Med.

[19] Farkas H, Harmat G, Kaposi PN, Karádi I, Fekete B, Füst G (2001). Ultrasonography in the diagnosis and monitoring of ascites in acute abdominal attacks of hereditary angioneurotic oedema. Eur J Gastroenterol Hepatol.

[20] Bork K, Staubach P, Eckardt AJ, Hardt J (2006). Symptoms, course, and complications of abdominal attacks in hereditary angioedema due to C1 inhibitor deficiency. Am J Gastroenterol.

[21] Zuraw BL, Herschbach J (2000). Detection of C1 inhibitor mutations in patients with hereditary angioedema. J Allergy Clin Immunol.

[22] Bowen B, Hawk JJ, Sibunka S, Hovick S, Weiler JM (2001). A review of the reported defects in the human C1 esterase inhibitor gene producing hereditary angioedema including four new mutations. Clin Immunol.

[23] Bork K (2013). Hereditary angioedema with normal C1 inhibitor. Immunol Allergy Clin North Am.

[24] Bork K, Wulff K, Witzke G, Hardt J (2015). Hereditary angioedema with normal C1-INH with versus without specific F12 gene mutations. Allergy.

[25] Giavina-Bianchi P, Aun MV, Motta AA, Kalil J, Castells M (2015). Classification of angioedema by endotypes. Clin Exp Allergy.

[26] Quincke HI (1982). Über akutes umschriebenes Hautödem. Monatsh Prakt Dermatol.

[27] Giavina-Bianchi P, Aun MV, Jares EJ, Kalil J (2016). Angioedema associated with nonsteroidal anti-inflammatory drugs. Curr Opin Allergy Clin Immunol.

[28] Aun MV, Blanca M, Garro LS, Ribeiro MR, Kalil J, Motta AA (2014). Nonsteroidal anti-inflammatory drugs are major causes of drug-induced anaphylaxis. J Allergy Clin Immunol Pract.

[29] Osler W (1888). Hereditary angioneurotic edema. Am J Med Sci.

[30] Gomes E, Cardoso MF, Praça F, Gomes L, Mariño E, Demoly P (2004). Self-reported drug allergy in a general adult Portuguese population. Clin Exp Allergy.

[31] Stevenson DD, Sanchez-Borges M, Szczeklik A (2001). Classification of allergic and pseudoallergic reactions to drugs that inhibit cyclooxygenase enzymes. Ann Allergy Asthma Immunol.

[32] Kowalski ML, Asero R, Bavbek S, Blanca M, Blanca-Lopez N, Bochenek G (2013). Classification and practical approach to the diagnosis and management of hypersensitivity to nonsteroidal anti-inflammatory drugs. Allergy.

[33] Landerman NS, Webster ME, Becker EL, Ratcliffe HE (1962). Hereditary angioneurotic edema. II. Deficiency of inhibitor for serum globulin permeability factor and/or plasma kallikrein. J Allergy.

[34] Donaldson VH, Evans RR (1963). A biochemical abnormality in herediatry angioneurotic edema: absence of serum inhibitor of C'1-esterase. Am J Med.

[35] Rosen FS, Pensky J, Donaldson V, Charache P (1965). Hereditary angioneurotic edema: two genetic variants. Science.

[36] Dunkelberger JR, Song WC (2010). Complement and its role in innate and adaptive immune responses. Cell Res.

[37] Ratnoff OD, Pensky J, Ogston D, Naff GB (1969). The inhibition of plasmin, plasma kallikrein, plasma permeability factor, and the C'1r subcomponent of the first component of complement by serum C'1 esterase inhibitor. J Exp Med.

[38] Bork K, Witzke G, Artmann K, Benes P, Böckers M, Kreuz W (1984). Interaction between C1-INA, coagulation, fibrinolysis and kinin system in hereditary angioneurotic edema (HANE) and urticaria. Arch Dermatol Res.

[39] Walford HH, Zuraw BL (2014). Current update on cellular and molecular mechanisms of hereditary angioedema. Ann Allergy Asthma Immunol.

[40] Rocha e Silva M, Beraldo WT, Rosenfeld G (1949). Bradykinin, a hypotensive and smooth muscle stimulating factor released from plasma globulin by snake venoms and by trypsin. Am J Physiol.

[41] Elliott DF, Horton EW, Lewis GP (1960). Actions of pure bradykinin. J Physiol.

[42] Venema VJ, Marrero MB, Venema RC (1996). Bradykinin-stimulated protein tyrosine phosphorylation promotes endothelial nitric oxide synthase translocation to the cytoskeleton. Biochem Biophys Res Commun.

[43] Nussberger J, Cugno M, Amstutz C, Cicardi M, Pellacani A, Agostoni A (1998). Plasma bradykinin in angio-oedema. Lancet.

[44] Nussberger J, Cugno M, Cicardi M (2002). Bradykinin-mediated angioedema. N Engl J Med.

[45] Nussberger J, Cugno M, Cicardi M, Agostoni A (1999). Local bradykinin generation in hereditary angioedema. J Allergy Clin Immunol.

[46] Han ED, MacFarlane RC, Mulligan AN, Scafidi J, Davis AE (2002). Increased vascular permeability in C1 inhibitor-deficient mice mediated by the bradykinin type 2 receptor. J Clin Invest.

[47] Cicardi M, Banerji A, Bracho F, Malbrán A, Rosenkranz B, Riedl M (2010). Icatibant, a new bradykinin-receptor antagonist, in hereditary angioedema. N Engl J Med.

[48] Cicardi M, Levy RJ, McNeil DL, Li HH, Sheffer AL, Campion M (2010). Ecallantide for the treatment of acute attacks in hereditary angioedema. N Engl J Med.

[49] Dewald G, Bork K (2006). Missense mutations in the coagulation factor XII (Hageman factor) gene in hereditary angioedema with normal C1 inhibitor. Biochem Biophys Res Commun.

[50] Schmaier AH (2016). The contact activation and kallikrein/kinin systems: pathophysiologic and physiologic activities. J Thromb Haemost.

[51] Cichon S, Martin L, Hennies HC, Müller F, Van Driessche K, Karpushova A (2006). Increased activity of coagulation factor XII (Hageman factor) causes hereditary angioedema type III. Am J Hum Genet.

[52] Björkqvist J, de Maat S, Lewandrowski U, Di Gennaro A, Oschatz C, Schönig K (2015). Defective glycosylation of coagulation factor XII underlies hereditary angioedema type III. J Clin Invest.

[53] de Maat S, Björkqvist J, Suffritti C, Wiesenekker CP, Nagtegaal W, Koekman A (2016). Plasmin is a natural trigger for bradykinin production in patients with hereditary angioedema with factor XII mutations. J Allergy Clin Immunol.

[54] Iahn-Aun M, Aun MV, Motta AA, Kalil J, Giavina-Bianchi P, Hayashida SA (2017). The complex interaction between polycystic ovary syndrome and hereditary angioedema: case reports and review of the literature. Obstet Gynecol Surv.

[55] Citarella F, Misiti S, Felici A, Aiuti A, La Porta C, Fantoni A (1993). The 5' sequence of human factor XII gene contains transcription regulatory elements typical of liver specific, estrogen-modulated genes. Biochim Biophys Acta.

[56] Madeddu P, Emanueli C, Song Q, Varoni MV, Demontis MP, Anania V (1997). Regulation of bradykinin B2-receptor expression by oestrogen. Br J Pharmacol.

[57] Chen LM, Chung P, Chao S, Chao L, Chao J (1992). Differential regulation of kininogen gene expression by estrogen and progesterone in vivo. Biochim Biophys Acta.

[58] Matesic D, Fernández Pérez ER, Vlahakis NE, Hagan JB (2006). Acute pancreatitis due to hereditary angioedema. Ann Allergy Asthma Immunol.

[59] Li HH, Busse P, Lumry WR, Frazer-Abel A, Levy H, Steele T (2015). Comparison of chromogenic and ELISA functional C1 inhibitor tests in diagnosing hereditary angioedema. J Allergy Clin Immunol Pract.

[60] Wagenaar-Bos IG, Drouet C, Aygören-Pürsün E, Bork K, Bucher C, Bygum A (2008). Functional C1-inhibitor diagnostics in hereditary angioedema: assay evaluation and recommendations. J Immunol Methods.

[61] Farkas H, Jakab L, Temesszentandrási G, Visy B, Harmat G, Füst G (2007). Hereditary angioedema: a decade of human C1-inhibitor concentrate therapy. J Allergy Clin Immunol.

[62] Weiler CR, van Dellen RG (2006). Genetic test indications and interpretations in patients with hereditary angioedema. Mayo Clin Proc.

[63] Caballero T, Farkas H, Bouillet L, Bowen T, Gompel A, Fagerberg C (2012). International consensus and practical guidelines on the gynecologic and obstetric management of female patients with hereditary angioedema caused by C1 inhibitor deficiency. J Allergy Clin Immunol.

[64] Kiss N, Barabás E, Várnai K, Halász A, Varga LÁ, Prohászka Z (2013). Novel duplication in the F12 gene in a patient with recurrent angioedema. Clin Immunol.

[65] Bork K, Wulff K, Meinke P, Wagner N, Hardt J, Witzke G (2011). A novel mutation in the coagulation factor 12 gene in subjects with hereditary angioedema and normal C1-inhibitor. Clin Immunol.

[66] Farkas H, Veszeli N, Kajdácsi E, Cervenak L, Varga L (2016). “Nuts and Bolts” of Laboratory Evaluation of Angioedema. Clin Rev Allergy Immunol.

[67] Zuberbier T, Aberer W, Asero R, Bindslev-Jensen C, Brzoza Z, Canonica GW (2014). The EAACI/GA(2) Len/EDF/WAO Guideline for the definition, classification, diagnosis, and management of urticaria: the 2013 revision and update. Allergy.

[68] Miller DR, Oliveria SA, Berlowitz DR, Fincke BG, Stang P, Lillienfeld DE (2008). Angioedema incidence in US veterans initiating angiotensin-converting enzyme inhibitors. Hypertension.

[69] Kostis JB, Kim HJ, Rusnak J, Casale T, Kaplan A, Corren J (2005). Incidence and characteristics of angioedema associated with enalapril. Arch Intern Med.

[70] Beltrami L, Zanichelli A, Zingale L, Vacchini R, Carugo S, Cicardi M (2011). Long-term follow-up of 111 patients with angiotensin-converting enzyme inhibitor-related angioedema. J Hypertens.

[71] Makani H, Messerli FH, Romero J, Wever-Pinzon O, Korniyenko A, Berrios RS (2012). Meta-analysis of randomized trials of angioedema as an adverse event of renin-angiotensin system inhibitors. Am J Cardiol.

[72] Schreiber AD, Zweiman B, Atkins P, Goldwein F, Pietra G, Atkinson B (1976). Acquired angioedema with lymphoproliferative disorder: association of C1 inhibitor deficiency with cellular abnormality. Blood.

[73] Mandle R, Baron C, Roux E, Sundel R, Gelfand J, Aulak K (1994). Acquired C1 inhibitor deficiency as a result of an autoantibody to the reactive center region of C1 inhibitor. J Immunol.

[74] Zingale LC, Castelli R, Zanichelli A, Cicardi M (2006). Acquired deficiency of the inhibitor of the first complement component: presentation, diagnosis, course, and conventional management. Immunol Allergy Clin North Am.

[75] Zuraw BL, Bernstein JA, Lang DM, Craig T, Dreyfus D, Hsieh F (2013). A focused parameter update: hereditary angioedema, acquired C1 inhibitor deficiency, and angiotensin-converting enzyme inhibitor-associated angioedema. J Allergy Clin Immunol.

[76] Zuraw BL, Curd JG (1986). Demonstration of modified inactive first component of complement (C1) inhibitor in the plasmas of C1 inhibitor-deficient patients. J Clin Invest.

